# ADAPTED CONVERSATION ANALYSIS TO EVALUATE DYADIC DEMENTIA-RELATED ADVANCED CARE PLANNING DISCUSSIONS

**DOI:** 10.1093/geroni/igae098.3567

**Published:** 2024-12-31

**Authors:** Sara Bybee, Rebecca Utz, Jordana Clayton, Nancy Aruscavage, Katherine Supiano, Eli Iacob, Kara Dassel

**Affiliations:** University of Utah College of Nursing, Salt Lake City, Utah, United States; University of Utah, Salt Lake City, Utah, United States; University of Utah, Salt Lake City, Utah, United States; University of Utah, Salt Lake City, Utah, United States; University of Utah, Salt Lake City, Utah, United States; University of Utah, Salt Lake City, Utah, United States; University of Utah, Salt Lake Cty, Utah, United States

## Abstract

Persons living with dementia (PLWD) and their care partners should engage in advance care planning (ACP) prior to serious cognitive decline. This pilot study assessed the effectiveness of the Life-Planning in Early Alzheimer’s and Other Dementias (LEAD) Guide in promoting ACP concordance among PLWD and their care partners. Discussions of dyads’ (N=7) responses to the LEAD Guide were audio/video-recorded and analyzed by two coders for the following elements of Conversation Analysis (CA) (related to ACP concordance and relationship quality, respectively): turn-taking (listening leading to understanding, respect/mutual regard); Repair (giving and taking to gain shared understanding, apology); Action formation (assumed topic of discussion and norms, agreement on discussion subject and scope); Action ascription (trusting, understanding of previous turn); Action sequencing (acceptance of discussion norms towards goal, unconditional love). Coders demonstrated moderate agreement across all recordings (M=73.1%, SD=4.4), with unanimous agreement on: the presence of listening leading to understanding, assumed topic of discussion and norms, agreement on the subject and scope of discussion, and understanding of previous turn in all recordings, and the absence of apology and blind trust in 3 (42.8%) recordings. CA may be unable to capture ACP concordance as some elements may be inherent to the LEAD Guide discussion itself while others are unobservable in ACP conversations. To objectively examine how interactions foster ACP concordance, CA methodology should account for 1) multiple ACP discussions, 2) factors related to either the LEAD Guide or dementia diagnosis that impact turn-taking, and 3) the dynamics between the PLWD and their care partner.

